# Treatment of Enlarged Prostate

**Published:** 1878-08

**Authors:** 


TREATMENT OF ENLARGED PROSTATE.—Dr. Washington L. Atlee, in an article to the *Medical and Surgical Reporter* of the 18th inst., on enlarged prostrate, suggests a plan of treatment which he bases on the three following propositions:

1. That the prostate and its vessels are possessed of unstriped muscular fibre.

2. That the bladder is a hollow organ, with an involuntary muscular coat.

3. That ergot will contract unstriped or involuntary muscular tissue, as it does in the uterus.

Therefore, as a corollary, ergot ought to be a remedy for enlarged prostate and its effects.

This was the theory on which I based the practice, and whether the rationale is correct or not, my experience in the use of ergot in such cases has been most satisfactory. Several patients over sixty years of age have been treated with ergot, and have been able to lay aside the catheter after having been the victims of its daily use. When called to a case of retention from enlarged prostate, my rule is first to relieve the bladder by means of the catheter, and follow this immediately by ordering twenty drops of the fluid extract of ergot every four hours, until the patient gets entire control over his bladder. Until this is accomplished, I continue to relieve him with the catheter every twelve hours. As his power of urination is restored I diminish the frequency of the medicine, and gradually end in giving a dose every night. A gentleman who died last month, at the age of ninety-two, was exceedingly ill in August, 1872, in consequence of retention of urine from enlarged prostate, and had to be regularly catheterized for relief. He was placed upon the above treatment, and in a few days was able to do without his catheter. His urinary organs were kept in good condition by taking a dose of ergot every night, and he enjoyed much better health in consequence, and died recently, of old age. I mention this case in particular, because a post-mortem examination proved to me that the prostate had been diminished in size by the treatment.

In these cases it is very common for sedimentary deposits to accemulate in the bladder, which become a source of irritation and discomfort, and if the organ should fail to expel its contents entirely, it is the best every few days to introduce the catheter to remove them.

